# A Review of the Epidemiological Methods Used to Investigate the Health Impacts of Air Pollution around Major Industrial Areas

**DOI:** 10.1155/2013/737926

**Published:** 2013-06-02

**Authors:** Mathilde Pascal, Laurence Pascal, Marie-Laure Bidondo, Amandine Cochet, Hélène Sarter, Morgane Stempfelet, Vérène Wagner

**Affiliations:** French Institute for Public Health Surveillance, 12 rue du Val d'Osne, 94 415 Staint-Maurice, France

## Abstract

We performed a literature review to investigate how epidemiological studies have been used to assess the health consequences of living in the vicinity of industries. 77 papers on the chronic effects of air pollution around major industrial areas were reviewed. Major health themes were cancers (27 studies), morbidity (25 studies), mortality (7 studies), and birth outcome (7 studies). Only 3 studies investigated mental health. While studies were available from many different countries, a majority of papers came from the United Kingdom, Italy, and Spain. Several studies were motivated by concerns from the population or by previous observations of an overincidence of cases. Geographical ecological designs were largely used for studying cancer and mortality, including statistical designs to quantify a relationship between health indicators and exposure. Morbidity was frequently investigated through cross-sectional surveys on the respiratory health of children. Few multicenter studies were performed. In a majority of papers, exposed areas were defined based on the distance to the industry and were located from <2 km to >20 km from the plants. Improving the exposure assessment would be an asset to future studies. Criteria to include industries in multicenter studies should be defined.

## 1. Introduction

Industrial areas are characterized by a high density of industries, sharing common infrastructures, such as transport networks, waste water treatment plants, and waste incineration plants. These areas cluster at-risk activities and pollution sources. They have historically attracted, and may still attract, hundreds of employees who have settled in the vicinity of the plants. With extensive urbanization, industrial areas have been embedded in the urban landscape, increasing the nuisances and the exposure of the population. For instance, in the South of France, the industrial area of l'etang de Berre hosts 430 industries classified for the protection of the environment and more than 60% of the Seveso II (referring to the European directive 96/82/CE) plants of the region. About 16 towns representing more than 300,000 inhabitants are exposed to the plumes produced by these plants [1]. 

People living near major industrial areas are facing complex situations of exposure: occupational and environmental exposure, multiexposure to chemicals combined with exposure to noise, dusts, visual pollution, stress, and so forth The possible associated health risks are of the highest concern to the population. 

Quantitative health risk assessments, based on the comparison of a hypothetical exposure (assessed through measured or modeled concentrations in different matrices combined with scenarios of exposure) to toxicological reference values or to regulatory values, have been extensively used for regulatory purposes. They can point out problems with specific pollutants or route of exposure. For instance, several risk assessments around large French industrial areas found that the levels of some compounds, including benzene, particulate matter (PM), and SO_2_, could be considered too high [2, 3]. They confirmed that the concerns of the population were legitimate and triggered actions to reduce those specific pollutions. Yet, quantitative health risk assessments can neither tell if and how many people are actually suffering because of the pollution, nor they can take into account the integrated burden of the multiexposure to the chemical, physical, and perceived industrial pollution. The answers to these questions belong to epidemiologists and raise several methodological issues: what kind of study should be used, which health outcomes should be investigated, how to assess exposure, and how to control for confounding factors?

In this paper, we performed a literature review of the published studies investigating the health of population exposed to industrial air pollution around major industrial sites. The objectives were (1) to identify the reasons why studies were performed, (2) to list the health outcomes that have been investigated, (3) to describe the study designs that have been used, and (4) to describe and discuss the exposure assessments. The objectives were not to perform a systematic review but to collect a representative sample of the different practices that can be used in that field.

## 2. Methods

The work focused on studies investigating the chronic effects of air pollution from large industrial areas and major complexes grouping several plans or multicenter studies involving similar types of industry that could be or not part of larger industrial complexes. 

Papers published between 1980 and 2012 were searched based on the Scopus database that includes PubMed and other relevant literature database. As an initial research using key words referring to industry retrieved very few papers, we searched epidemiological studies on the impacts of air pollution around point sources. On a second step, papers dealing with industries were selected based on the title and abstracts.

The initial search equation was ((“Air Pollutants” [MeSH] OR “Air Pollution” [MeSH]) AND “epidemiology” [Subheading]) OR ((Air pollution [Title/Abstract] OR Air pollutants [Title/Abstract]) AND (epidemiology OR epidemio* OR “Case control study” OR cohort OR “cross sectional study” OR prevalence OR incidence OR Surveillance OR survey OR “Health risk” OR “Risk assessment” OR health OR “Health effects” OR Exposure OR “Health impact*” OR Mortality OR “Adverse effects”)) AND (industry OR industrial) AND (residents OR Residential OR inhabitants OR neighborhood* OR vicinity OR “living area” OR “living near” OR surrounding* OR populations).

Papers were analyzed focusing on the types of industries, the study design, the health indicators, and the exposure assessment. The objectives were to identify the methods but not to discuss the results reported by each paper. To do so, reviews focusing on specific industries would be more relevant. 

## 3. Results

From the initial search 230 papers in English or French were selected based on their title. Based on the abstracts, 155 papers were excluded (58 environmental studies only, 35 looking at exposure through water, soil, or food and not air directly, 36 using industrial areas as one source among other air pollution sources, 10 description of the health of a population without links to exposure, 8 on nuclear installations, 4 toxicological studies, 3 studies focusing on acute exposure after an accident, and 1 literature review). Two reports from the grey literature were added, but no specific search was performed to identify such reports on a larger scale.

77 papers were finally included in the review, published between 1989 and 2011. While papers were available from many different countries, a majority of papers came from 3 European countries: the United Kingdom, Italy, and Spain (Table 1). One paper may provide results for several studies, and 27 studies were focusing on cancer, 25 on morbidity, 9 on biomonitoring, 7 on mortality or birth outcome, and 3 on mental health. Studies for each of these health outcomes are described below.

### 3.1. Cancers

#### 3.1.1. Reasons for Performing Studies on Cancers

The 27 studies on cancer are detailed in Table 2. 12 studies were multicenter studies, ranging from 4 sites to 452 sites.

The reasons for doing an epidemiological study on cancer near a major industrial area were frequently concerns from the population, explicitly quoted by 7 studies [1, 7, 12, 15, 17, 25, 27]. Few studies gave details on the social background, showing that the health issues crystallized the concerns of the population. For instance, Bhopal et al. states that *“ *[⋯]* the controversy was such that the health concerns were central issues in a public inquiry, and received extensive media coverage. Our study was requested by the local authority, to help resolve this controversy”. *Sans et al. reports that their “*study was undertaken in response to concerns of a local pressure group based *[⋯]* about an alleged cluster of cancer, especially of the larynx, and leukaemia among children *[⋯]* there was also concern about several deaths among teachers and pupils at the nearby comprehensive school”. *In 11 other studies, concern of the population is not mentioned, but the authors justified their study with references to an overincidence of cancers or mortality observed in the area by previous investigation [4–6, 8, 9, 14, 16, 19, 26, 28, 31]. 

By contrast, multicenter studies refer to the literature and possible etiology in relation to the emissions to justify their choices [20–22, 24], although geographical variations of the incidence of the cancers investigated are also used as a justification for focusing in a specific region or on a specific cancer [10, 18, 23]. 

#### 3.1.2. Industries Involved in the Studies on Cancers

Study areas vary from very rural areas with about 2,000 inhabitants [17] to highly populated areas with several hundred thousand people [1, 4, 12]. The industries involved in the studies are highly heterogeneous and usually have been operating since several decades before the study period, with areas sometimes industrialized since the 19th century. Six studies were on refineries [6, 16, 17, 26, 28], including one multicenter study in the United Kingdom [14], and 3 on petrochemical plants [15, 27], including one multicenter study in Louisiana [7]. Larger sites gather a variety of different industries. For instance, Teesside includes iron, steel industries, chemical, and heavy engineering industries. By 1945 it was the largest single chemical production complex in the world [8]. In France, a site like Etang de Berre involves oil refining, oil storage, petrochemical and organic chemical activities, chlorine chemistry, steel and metal working, waste incineration plant, and the port for ore and oil tankers [1].

Among the multicenter studies, industrial sites of different natures were involved in a study in Italy [4, 5] and in Spain [24]. Wilkinson et al. studied 11 petrol refineries corresponding to 7 industrial areas [14]. In a study investigating the petrochemical industries in Louisiana, Simonsen et al. used three different criteria to aggregate the industries: (1) all sites were considered as a whole, without regard to specific emissions; (2) sites were classified on the basis of their Standard Industrial Classification code as either belonging to the petrochemical industry or not; (3) sites were classified on the basis of the International Agency for Research on Cancer (IARC) carcinogen rating assigned to their specific chemical releases [7].

European registries of polluting industries were extensively used in Spain [10, 18, 20–24, 32, 33] to perform the multicenter studies. In some cases [10, 23, 24], all sites were included. For instance, the study by Cambra et al. included 66 sites, aggregated into 6 categories: 4 energy production plants, 28 metalworking industries, 8 cement industries, 44 chemical industries, and 17 others [24]. In other cases, only the industries corresponding to one activity, for example, metal production [20, 22] or paper, pulp, and board industries [18], were included. 

#### 3.1.3. Type of Studies Investigating Cancers

Most of the studies (20/27) used a geographical ecological design, based on standardized mortality or morbidity ratios, searching for a possible overincidence of the mortality or the morbidity. Poisson regression and similar statistical designs were used to assess a relationship between health indicators and exposure, taking into account confounding factors (mostly socioeconomic) (Table 2). 

Seven studies were case-control studies [4–10]. For instance, Zambon et al. included 172 cases of sarcoma and 405 controls in their study [4]. Biggeri et al. collected data from 755 cases of lung cancer and 755 controls [5]. The multicenter design was used either for case-control studies [4, 5, 7, 10] or for standardized incidence or mortality ratio studies [14, 18, 20–24, 31]. 

Lung cancer was the most commonly studied [1, 5, 7–10, 15, 16, 18, 19, 21, 24, 25, 28, 34], based on registries, mortality data, or hospitalizations data [10]. Other cancers investigated were leukemia [6, 15, 20, 25–27], digestive cancers [22], non-Hodgkin's lymphoma [23] and sarcoma [4], either based on mortality or registry data. 

The latency of cancer was usually taken into account as the number of years of residence in the area before deaths. It varied from at least 1 year (e.g., [9]) to 10 years (e.g., [8]) and was sometime unspecified.

#### 3.1.4. Exposure Assessment in the Studies Investigating Cancers

Distance was used as the method to assess the exposure in 19 of the studies. The use of distance is seen as a way to overcome the lack of measurement data, but also to reduce the latency problem, as clearly stated by Pless-Mulloli et al.:* “areas closest to steel and chemical plants at the time of study were also close 40 years earlier, an important consideration given the long latency of lung cancer”* [19]. However, this requires the assumption that people were also living in the same area 40 years earlier.

Several options were used for the distance (Table 2), for instance,

  exposed group (“near”) ≤ 5 km from a metal production plant, intermediate ≤ 5 km from any industrial installation other than metal production and processing, unexposed group (“far”), consisting of towns having no EPER-registered industry within 5 km of their municipal centroid (reference level) [18],
  distance: 0–2 km, 0–7.5 km, and eight bands around refinery perimeters with outer limits at 0.5, 1, 2, 3, 4.5, 5.6, 6.6, and 7.5 km [14],
  three concentric circles with radii of 3, 8, and 10 km for descriptive purposes and 10 concentric circles with a radius increasing from 1 to 10 km to define nine bands [25].

Additional refinement may be added, taking into account, for instance, the residential history [7]. Bhopal et al. made an original combination of different metrics to characterized exposure: perceived exposure areas (criteria not specified), modeled exposure (model not specified), and the 24-hour mean daily measures of SO_2_ and smoke over 56 months [12]. In Finland, the exposure area was based on distance, but that distance was chosen based on measurements of sulfuric acid and the prevailing wind directions [9]. Edwards et al. also mentioned that their choice of the distance was guided by a validation study using data from historical records and measurements [8].

Another example of a complex exposure assessment initially relying on distance is given by Yu et al.: to account for the effects from monthly prevailing wind, they defined exposure wedges for each month by the monthly prevailing wind direction. Only addresses located within the exposure wedges were considered exposed during the particular month, and the exposure opportunity scores for these residences were assigned by the inverse of distance to the relevant petrochemical complexes [6].

Although reference sites are usually defined as the farthest to the plant, some studies include a further subclassification taking into account proximity to traffic, urban, semiurban, and rural areas. The definition of these areas may vary between studies. For instance, the industrial area can be defined based on the distance between the subject's residence and an industrial installation (industrial distance), as the area defined by the first decile of industrial distance [10]. 

Models were used by only 5 studies. The Industrial Source Complex Model in long-term model was used by Zambon et al. [4], and Atmospheric Dispersion Model System version 3-ADMS 3 was used in France [1, 31]. The other two models were not detailed [12, 15]. In the Etang de Berre study, results from the models were combined with measurements to obtain a map of the annual mean levels of SO_2_, which were then grouped in three classes of exposure based on quartiles [1]. Viel et al. derived two indicators from the air pollution model, corresponding to different hypotheses about the mode of exposure: the concentrations alone represented exposure from inhalation only; the number of years the plant had operated and the degradation speed in soils provided a cumulative ground-level concentrations since the start of the activity [31].

The lack of emission data is a key limitation to modeling, acknowledged by some authors [16]. In France, Viel et al. used a complex process to recreate emissions based on exposure judgment in order to be able to complete the dispersion modeling [31]. 

Measures alone were used by one study only, taking advantage of a relatively dense air quality monitoring network for SO_2_ and PM_10_ [16]. More frequently, measures were used to describe areas previously chosen based on distance or modeling, and measurements were not input in the statistical models. For instance, in the case of Stenungsund in Sweden, models (unspecified) of ethylene levels based on the emissions of year 2000 were used to classify a low and a high exposure area. Measurements were performed in the high exposure areas (ethylene, propylene, benzene, 1,3-butadiene, 1,2-dichloroethane (EDC), and vinyl chloride) in 2001-2002 and 2006-2007. They were used to perform ahealth risk assessment but not directly in the epidemiological study [15]. In the area of Teesside, abundant routinely available air quality data, *“reflecting long standing concerns about air pollution there,”* [13] were used to check the validity of the selection of study areas based on residential proximity to industry as a proxy for exposure [13].

### 3.2. Morbidity

Studies on morbidity are detailed in Table 3. Again, there is a great diversity of the industries involved in the studies, similar to those described for cancer. 

#### 3.2.1. Reasons for Performing Studies on Morbidity

Concern was a major motivation quoted by 12 studies [1, 12, 35, 36, 38, 41, 46, 48, 51, 54, 57]. For instance, Bhopal et al. stated that *“one of the major concerns among the residents *[⋯]* was an apparent increase in the incidence of asthma in the area” *[12]. Reference to previous studies showing over-incidences of cancer, mortality or asthma are also quoted by 11 studies [37, 40, 42, 45, 48, 49, 52, 53, 56]. For instance, in the area investigated by Halliday et al*., “the prevalence of childhood asthma *[⋯]* was approximately twice that of a control area *[⋯]*”* [42]. One study mentioned that an acute episode had severe impacts, resulting in hospitalizations [57]. 

#### 3.2.2. Health Outcome and Type of Studies Investigating Morbidity

A majority of the studies focused on the respiratory health of children (17 studies), using questionnaires specifically defined for the study or standardized questionnaires such as the ISAAC questionnaire from the International Study of Asthma and Allergies in Childhood [39, 45, 47, 54], or the questionnaire from the American Thoracic Society (ATS) [40, 43]. Few studies used additional data from general practitioners (GPs) [12, 13, 49, 59]. Studies involved from 200 to 500 children [41, 43, 47] to more than 3000 children [59]. 6,399 adults were also interviewed in Teesside [12], while in India the respiratory health of 2573 women was investigated [38]. Several studies also involved measurements of the lung function. One study in Thailand investigated short-term memory dysfunction in children through questionnaires [57] (Table 3). One study focused on odor annoyance, based on the observation that *“odors from industrial sources, such as the petrochemical plants in Sarnia, have been shown to considerably impact general health and well-being by affecting both the physiological and psychosocial status of people”* [58]. 

#### 3.2.3. Type of Studies Investigating Morbidity

Two studies were intervention studies. Câra et al. compared GPs information on the respiratory health of 874 children for two periods: when the industry was operating and after its closure [49]. Stenlund et al. investigated the influence of a measure taken to reduce air pollution (predominantly dust and soot) on perceived pollution, risk perception, annoyance, and health symptoms through interviews of 684 people [46].

Five studies used an ecological approach to study standard rates ratio based on hospital admissions or disease incidence. Two studies quantified the relationship between symptoms and measurements through a time-series analysis [12] and a case-cross over analysis [52]. 

#### 3.2.4. Exposure Assessment in the Studies Investigating Morbidity

Participants of the cross-sectional surveys were selected based on their city of residence (or school), and distance was again the preferred method to define the exposed versus nonexposed cities. In most studies, a finer exposure assessment was performed for the participants, based on information collected through the questionnaires, modeling, or measurements. When measurements were available, they were not always used to assess exposure. For instance, Moraes et al. mentioned that concentrations were available for several pollutants (PM, NOx; SO_2_, O_3_, benzene, toluene, and xylenes) but used them for descriptive purposes only (in comparison to the World Health Organization air quality standards) [47].

White et al. reported that they did not have the budget for a model and that concentration and emissions data were missing. Therefore, they add that they rely on a meteorologically estimated exposure index based on wind direction and speed [54]. Aylin et al. also explained that they had to use distance because input data for the dispersion modeling were missing [37].

Fung et al. selected the participating cities based on the annual averages of SO_2_, NO_2_, and O_3_ and mentioned that the reference area *“is polluted but considered ‘clean' compared to the two more polluted other cities*” [35].

Pless-Mulloli et al. proposed two indicators to characterize the long-term versus short-term exposure: short-term exposure was assessed through PM_10_ measurements, and long-term exposure was defined as living near an active site [59]. Regarding short-term, acute exposure, Dubnov et al. developed a complex indicator for episodes when NOx and SO_2_ concentrations were high. For each episode, they computed an integrated concentration value (ICV) as NOx multiplied by SO_2_ summarized the results over the entire study period (3 years) [40]. 

One study compared the associations between emergency department visits and SO_2 _concentrations obtained from fixed monitors and from an air dispersion modeling and found some differences increasing with the distance [53].

#### 3.2.5. Mortality (from Other Causes Than Cancer)

Studies on mortality are detailed in Table 4. They were all geographical ecological studies, distance being used as the exposure indicator except in one study relying on SO_2_ dispersion modeling [60]. Sarov et al. investigated perinatal mortality and used odors complaints to define the distance [61]. One study was multicentric, focusing on 10 coke works operating in England and listed in the Coke Oven Managers Association [62].

### 3.3. Birth Outcome

#### 3.3.1. Reasons for Performing Studies on Birth Outcome

Studies are summarized in Table 5. Seven studies on birth outcomes were identified, with three focusing on the same petrochemical area in Taiwan [28, 65, 66]. The main sites were those already investigated for other health issues, such as Teesside. Again, concerns of the population were the main reason for investigation in the studies focusing on a single area [12, 13, 67], while results from the literature and etiology were the reasons for the three multicenter studies [68–70]. In Taiwan, studies were justified on observed excess cancer mortality among women [28, 71]. 

#### 3.3.2. Type of Studies and Exposure Assessment in the Studies Investigating Birth Outcome

The health outcomes and the study design were various. Exposure assessment was poorly described compared to papers dealing with cancer or morbidity. Distance was the method used by all the studies but one [12], although extensive measurements were available in some sites, like in Israel, for instance [67]. In that case, the measurements and the wind rose were used to validate the choice of the distance, resulting in a large exposed area, up to 20 km. By contrast, in the multicenter study in Texas, proximity to industrial sites was defined at 1 mile or less [69]. 

### 3.4. Mental Health 

Three studies investigated mental health, psychological distress [72, 73], and one study investigated perceived pollution, perceived health and stigma [74]. All relied on postal questionnaires that may be complemented by a smaller number of semistructured face-to-face interviews [74]. For instance, the study by Bush et al. involved 5000 questionnaires and semi-structures in-depth interviews with 41 respondents. Participants were located in three areas distant to the site (1.5, 7, and 8 km) (Table 7).

#### 3.4.1. Reasons for Performing Studies on Mental Health and Perceived Health

The local background and concerns of the population were not the main motivation in the two studies in the United States based on industrial registries [72, 73]. On the contrary, population concern was a major issue in the study on Teesside [74], as stated by Bush et al., “*a place stigmatized not only for its heavy industry (technological stigma) but also on the basis of air pollution and poor health” *[74].

#### 3.4.2. Exposure Assessment for Performing Studies on Mental Health and Perceived Health

Two studies investigated the psychological distress of the population in relation to their proximity to industries registered in the Toxic Release Inventory through questionnaires. In these studies, the main assumption is not that an over-exposure to air pollutants can create adverse psychological effects, but that *“proximity to industrial activity is psychologically harmful because many individuals perceive industrial activity negatively, as a potential health threat or a sign of neighborhood disorder”* [73]. Therefore, exposure was defined based on distance, taking into account the volumes of the emissions as a proxy for facility size and visibility. The authors made the assumption that *“industrial facilities are not likely to impact residents' mental health if residents are unaware of them”* [72]. They propose a method to compute a potential visual exposure to industrial activity for each resident [72, 73]. 

### 3.5. Biomonitoring

Nine biomonitoring studies were reviewed. In none, even the one based in Teesside [77], concern of the population was mentioned as a motivation for the study. Participants were always recruited based on their residency in a city close to the industry. Additional data were usually collected to refine the exposure assessment of each participants for instance, near chlor alkali plant in Sweden and Italy, measurements of total gaseous mercury and a dispersion model (Transport Air Pollution Model (TAPM)) were used to assess the exposure at residence (Table 6) [75].

### 3.6. Results Described in the Studies

Discussing the results of the studies was not the objective of this literature review. However, it was interesting to note that when studying cancer, very few results were statistically significant, although several studies concluded on a gradient of risk following the exposure gradient [4, 19–21]. The risks estimated by the multicenter studies were also statistically nonsignificant, although significant risks may be found when a subanalysis of the study focuses on a single industry [18] or a subgroup of industries [23, 24]. 

Morbidity, and especially less severe outcomes such as respiratory symptoms, eyes symptoms or consultations to the general practitioners tended to increase with exposure [35, 39, 40, 42–45, 53–56, 62]. Similar results were found for hospitalizations for respiratory and cardiovascular causes [1, 34, 36, 52].

In the studies of declared health, complaints about odors or dust were correlated with the discomfort, in some cases positively [58] but also negatively [46]. The populations declaring a bad health status were not always the more exposed [13]. All studies on mental health underlined the influence of living near major industrial sites on psychological distress [72–74]. 

## 4. Discussion

### 4.1. Limits of the Literature Review

Epidemiological studies investigating the impacts of air pollution produced by major industrial sources are scarce, as only 77 papers were found in this review. They correspond to a wide range of industrial activities. However, our search is likely to be incomplete, and the limits of this search are probably the largest on the biomonitoring studies and the mental health studies, as we did not included these as explicit key words in the search.

However, given that the papers we included in the review were written by different teams, in different areas and at different periods, we are still confident that it can give a good overview of the practices in the field. Yet, it has to be noted that several papers were produced by the same team and/or part larger initiatives on industrial pollution, which may limit the diversity of practices reported. We also included two reports from the grey literature in the review [1, 36], but there are probably many unpublished work on the health status around industrial areas. For instance, Bentov et al. performed a study on the congenital malformation of a large industrial estate in Israel, explaining that their study was “*initiated by the Israel Ministry of Health, following complaints of residents of surrounding localities who blame the IP emissions for the odor nuisance and suspect that possible long- or short-term health disorders could be attributed to this exposure”* [67]. It is likely that other health outcomes have been investigated given the context, yet no paper was found on that area. Similarly, Rusconi et al. mentioned that an excess of respiratory symptoms in children was observed in the Sarroch region, near a major petrochemical area, referring to *“unpublished data”* [45]. 

Several reasons may explain the low number of publications; few epidemiological studies may be performed because of the complexity of collecting health and exposure data or because quantitative risk assessment is extensively used to study industrial pollution. There may also be a publication bias, with studies showing no link between exposure and health not being published. 

### 4.2. Site Selection and Studies Justification

In many of the cases, the studies are justified by a concern from the population; that is, epidemiology is used to test the hypothesis made by the population that the industries impair their health. It is also used to investigate areas where an overincidence of a health outcome had been previously observed. There are few initiatives to identify the health effects of a given industry independently of the local context, and these initiatives are mostly multicenter studies based on industrial registries indeed, whatever the topic (cancer, mental health, etc). 

In summary, the multicenter studies based on industrial registries are not taking into account the local context to select the areas under investigation, while mostly all others studies do. Therefore, there is likely to be a bias in site selection where to perform epidemiological studies, based on the existence of a local social mobilization. It would be interesting to understand why in some areas industries raised high concerns and lead to epidemiological studies, while in others there is such social mobilization, and if these reasons may result in biases in the result of the studies. On the other hand, it is essential to answer the population concerns, and, as stated by Ginns et al., *“the kind of epidemiological study we have conducted regards local concerns and beliefs as a ‘nuisance', the effect of an already sensitized population and an ‘obstacle to scientific enquiry' that seeks to uncover ‘real' health effects. A more socially informed epidemiology, however, would wish to give lay beliefs some prominence, to regard local concerns as data that are as valid as those derived from more formal questionnaires such as that used in the present study”. *A similar conclusion was reached by Phillimore on Teesside, showing that concern is an obstacle for epidemiology, especially when using questionnaires, as it introduces a bias in the population answer. But concern is also seen as an important issue by social scientists, including its possible health consequences [82, 83]. It is also interesting to note that several authors of the papers on mental health in these reviews are affiliated to social sciences department and that the papers were not published in epidemiological journals [72–74]. This calls for a broadening of the competency when answering the populations concerns near major industrial sites, that is, including a social sciences dimension in the analysis and not underestimating the influences of the industry and of its designation as a possible danger on the stress and well-being of the population.

### 4.3. Multicenter Studies

Multicentric design is believed to be a solution to the local biases, as the influence of the confounding factors may decrease as the number of sites increases [84]. However, it is difficult to identify relevant sites that could be included in the same studies. In the literature, the choices to aggregate industries based on large classes may hide differences linked to the industrial processes used, the size of the plant, its operating time, and so forth. Yet, multicenter studies may not fully answer the local concerns, and as Ramis stated, “*each industrial source has its own characteristics, and subsequent studies will therefore have to address these on a case-by-case basis”* [23].

### 4.4. Exposure Assessment

Independently of the health outcome and the statistical design used, the lack of information on the environmental and industrial background of the sites is striking in many papers. A major issue is raised by the exposure assessment. As industrial sites emit a complex mixture of pollutants, with plumes varying in composition and over time and space, epidemiologists have to rely on measurements and modeling of a subset of pollutants to assess an integrated exposure. Modeling is seen as the most efficient tool to avoid exposure misclassification. In Teesside, environmental data, land-use data, historical data, and data on the perception of air pollution and odors were analyzed to check that the distance to the site was an interesting proxy. Globally, measurements did not show large differences between exposed and nonexposed areas, but the dispersion models confirmed a gradient of pollution with distance [50]. However, environmental data and modeling are not easily accessed, especially when investigating past exposures. Indeed, several authors mentioned that emissions data were not available to perform a dispersion modeling or that they could not afford the cost of such modeling. Some authors underline that some environmental data collected for regulatory purposes are not usable for epidemiological studies [16]. 

This lack of environmental data is a major obstacle. It is striking to see that in many areas the population is highly concerned by the environmental pollution and its consequences, and that these concerns are answered through complex epidemiological studies, relying on poor environmental data. In short, there is a discrepancy between the expectancies of the population, the investment in collecting and analyzing health data, and the poor accessibility to key emissions and concentrations data. 

When distance is the only possible choice, Hodgson et al. advised to integrate knowledge of the factors that drive exposure, for example relative emissions, and wind direction [85]. Interestingly, odors are mentioned by several authors as an issue, but data are used to define the exposure area (e.g. [61]) and not to investigate a possible health impact.

The bias in exposure assessment and the ecological bias are likely to limit the possibility of ecological studies to reveal low relative risks with statistically significant results, especially when studying cancer with a latency of several decades. Leukemia may be the only cancer for which the latency is a priori short enough to allow a good reconstruction of exposure based on present data.

### 4.5. Ways Forward

A combination of multicentric studies and local studies could be efficient ways to increase knowledge on the health effects of industrial areas and answer the concerns from the population. As stated below, multicenter studies would limit local biases, and sites would not be selected based on an a priori population concern or over incidence. However, criteria to decide that sites are similar enough to be included in a multicenter study need to be defined. A focus on sites where the population requests more information could then be performed, with the support of social scientists. 

These studies could be performed on several health issues and with several designs. An investigation of the mental health impacts would be highly relevant, as this issue seems to have been poorly taken into account by epidemiologists so far. 

For the multicenter and the local studies, a better characterization of exposure would be an asset to improve our capacity to investigate the impacts of industrial pollution. It requires improving the availability of emission data and of monitoring data. 

Finally, intervention studies documenting the possible improvements of the health status of the population after the closure of a plant, or a change in the industrial processes, would be highly informative to improve the knowledge and to help for management (a change in the industrial processes that have been shown to have positive effect in the environment and the health status could be reproduced elsewhere).

## Figures and Tables

**Table 1:** Summary of the papers in the literature review.

Country	Total number of papers	Health outcome (several health outcomes may be described in 1 paper)					
		Cancer	Morbidity	Biomonitoring	Mortality	Birth outcome	Mental health
United Kingdom	15	5	5	2	4	0	1
Italy	9	3	3	2	1	0	0
Spain	8	7	0	1	1	0	0
Taiwan	7	4	0	0	0	3	0
Israel	6	1	3	0	8	1	0
United States	6	1	0	1	0	2	2
Canada	5	1	4	0	0	0	0
Sweden	4	1	1	2	0	0	0
France	2	2	1	0	0	0	0
Thailand	2	0	2	0	0	0	0

Countries with 1 study only		Finland, Lithuania	Argentina, Australia, Brazil, India, Romania, South Africa	Korea			

Total number of studies (several studies may be described in 1 paper)		27	25	9	7	7	3

**Table 2:** Studies investigating cancer.

Reference	Country	Industrial background	Health outcome	Epidemiological design	Exposure assessment
Zambon et al., 2007 [4]	Italy	Industrial waste incinerators, Municipal solid waste incinerators, Medical waste incinerators, thermal power plants, oil refinery industrial plants for the production of primary aluminium	Visceral and extravisceral sarcoma	Case control (72 cases and 405 controls)	Dispersion modeling (Industrial Source Complex Model in long-term mode, version 3 (ISCLT3))
Biggeri et al., 1996 [5]	Italy	Shipyard, iron foundry, incinerator, and Trieste city center	Lung cancer (mortality)	Case-control study (755 case-control pairs)	Distance and angle from each subject location to each pollution source
Yu et al., 2006 [6]	Taiwan	Oil refinery	Leukemia	Case control (171 cases and 410 controls)	Distance, based on previous studies (3 km radius from the geographic centroid of any of the four petrochemical complexes)
Simonsen et al., 2010 [7]	United States	Petrochemical industries	Lung cancer (registry)	Case control (455 cases and 437 controls)	Distance (0.5 miles, 1 mile, and 2 miles)
Edwards et al., 2006 [8]	United Kingdom	Iron and steel, chemical, and heavy engineering industries	Lung cancer (registry)	Case-control study (204 cases and 339 controls)	Distance, guided by a validation study using data from historical records
Petrauskaite et al., 2002 [9]	Lithuania	Production of mineral fertilizers, aluminum fluoride, and sulfuric acid	Lung cancer (mortality)	Case-control study (410 cases 410 controls)	Distance, based on measurements of sulfuric acid and the prevailing wind (6 km)
Lopez-Cima et al., 2011 [10]	Spain	23 industrial installations reporting to the EPER	Lung cancer	Case-control study (626 case, 626 controls)	Distance
Pascal et al., 2011 [1]	France	Oil refining, oil storage, petrochemical and organic chemical activities, chlorine chemistry, steel and metal working, chemical plants, waste incineration plant, port	All cancers, lung cancer, bladder cancer, breast cancer, multiple myeloma, malignant non-Hodgkin's lymphoma, and acute leukemia (hospitalisations)	Standardised incidence ratio	Coupling of a dispersion model (ADMS4), a meteorological model and kriging to assess the SO_2_ levels
Viel et al., 2011 [11]	France	13 municipal solid waste incinerators	Non-Hodgkin's lymphomas (registry)	Standardised incidence ratio	Dispersion model (Atmospheric Dispersion Model System version 3—ADMS 3) for each category of pollutants (dioxins, metals, and dusts)
					Perceived exposure areas (criteria not
Bhopal et al., 1994 [12] Bhopal et al., 1998 [13]	United Kingdom	Coke ovens (66 from 1980)	Cancer (registry)	Standardised incidence ratio	specified), modeled exposure (model not specified) 24-hour mean daily measures of SO_2_ and smoke over 56 months (1987–91)
Wilkinson et al., 1999 [14]	United Kingdom	11 oil refineries	Lymphohaematopoietic malignancy	Standardised incidence ratio	Distance (0–2 km, 0–7.5 km, and eight bands around refinery perimeters)
Axelsson et al., 2010 [15]	Sweden	Industrial complex including a large cracker producing ethylene and propene	Leukemia, lymphoma, cancers of the lung, liver, and central nervous system, all cancers taken together (registry)	Standardised incidence ratio	Models (unspecified) of ethylene levels
Eitan et al., 2010 [16]	Israel	Petroleum refineries, oil-fired power plant, and several large petrochemical, chemical, and agrochemical industries	Lung cancer, bladder cancer, and non-Hodgkin's lymphoma	Standardised incidence ratio	Spatial interpolation of SO_2_ and PM10 routine monitoring data
Schechter et al., 1989 [17]	Canada	Two natural gas refineries	Cancer (registry)	Standardised incidence ratio	Unclear
Monge-Corella et al., 2008 [18]	Spain	18 EPER-registered paper, pulp, and board industries	Lung cancer (mortality)	Standardised incidence ratio	Distance (≤5 km from a paper, pulp, and board industry, ≤5 km from any other industrial installation, towns having no EPER-registered industry within 5 km of their municipal centroid (reference level))
Pless-Mulloli et al., 1998 [19]	United Kingdom	Teeside	Lung cancer (mortality)	Standardised mortality ratio	Distance (0.1–2.7 km, 1.5–4 km, and farther)
García-Pérez et al., 2010 [20]	Spain	118 integrated pollution prevention and control (IPPC) category 2 metal production and processing installations which report their emissions to the EPER	Leukemia (mortality)	Standardised mortality ratio	See Monge-Corella
García-Pérez et al., 2009 [21]	Spain	57 combustion installations which report their emissions to the EPER	Lung, larynx, and bladder cancer (mortality)	Standardised mortality ratio	See Monge-Corella
García-Pérez et al., 2010 [22]	Spain	118 integrated pollution prevention and control (IPPC) category 2 metal production and processing installations that reported their releases to air and water in 2001	Tumours of the digestive system (mortality)	Standardised mortality ratio	See Monge-Corella
Ramis et al., 2009 [23]	Spain	452 industries reporting releases to air to the EPER, grouped by industrial sector	Non-Hodgkin's lymphomas (mortality)	Standardized mortality ratio	Distance (1, 1.5, and 2 km).
Cambra et al., 2011 [24]	Spain	284 industries declaring to the EPER emissions of pollutants	Lung cancer (mortality), haematological tumours (mortality)	Standardised mortality ratio	Distance (<2 km, >2 km)
Michelozzi et al., 1998 [25]	Italy	A large waste disposal site (one of the largest in Europe), a waste incinerator, and a petrochemical refinery	All cancers, laryngeal cancer, lung cancer, liver cancer, kidney cancer, and lymphatic and haematopoietic cancers (mortality)	Standardised mortality ratio	Distance (3, 8, 10 km, 10 concentric circles with a radius increasing from 1 to 10 km to define nine bands)
Pekkanen et al., 1995 [26]	Finland	Refinery	Leukemia, hematological cancers, all cancers (registries)	Standardised mortality ratio	Distance (4,4–7.9, 8–11.9, 12–15.9, and >16 km)
Sans et al., 1995 [27]	United Kingdom	Petrochemical processing: alcohols, styrene, olefins, benzene, vinyl chloride monomer, and polyvinyl chloride (PVC)	Cancer incidence and mortality for all cancers, leukaemias, and cancer of the larynx	Standardised mortality ratio	Distance (0–3 km, 7–5 km, and eight bands between circles of radii 0.5, 1–0, 2–0, 3–0, 4–6, 5–7, 6-7, and 7–5 km)
Yang et al., 2000 [28]	Taiwan	Kaohsiung oil refinery	Lung cancer (mortality)	Standardised mortality ratio	Distance
Pan et al., 1994 [29]	Taiwan	Kaohsiung oil refinery	Cancer in children (mortality)	Standardised mortality ratio	Distance
Tsai et al., 2009 [30]	Taiwan	Petrochemical industries	Bladder cancer (mortality)	Standardised mortality ratio	In each district, the number of employees of the industries divided by the population, in three clases

**Table 3:** Studies investigating morbidity.

Reference	Country	Industrial background	Health outcome	Epidemiological design	Exposure assessment
Fung et al., 2007 [35]	Canada	Sarnia “Chemical Valley”	All hospital admissions, admissions with a primary diagnosis of respiratory diseases and cardiovascular diseases	Standardized admissions ratio	Comparison of three cities, annual averages of SO_2_, NO_2_, and O_3_
Pascal et al., 2011 [1]	France	Oil refining, oil storage, petrochemical and organic chemical activities, chlorine chemistry, steel and metal working, chemical plants, waste incineration plant, port	Hospitalisations for cardiovascular and respiratory diseases	Poisson regression models	Coupling of a dispersion model (ADMS4), a meteorological model and kriging to assess the SO_2_ levels
Kosatsky et al., 2004 [36]	Canada	industrial area in Montreal	Hospitalisations for cardiovascular and respiratory diseases	Standardised admissions rates	O_3_, NO_*x*_, SO_2_, and PM measurements
Bhopal et al., 1994 [12] Bhopal et al., 1998 [13]	United Kingdom	Coke ovens (66 from 1980)	GPs activity: data on consultations, chronic conditions, hospital admissions, and current drug treatments. Lung function, Self-reported respiratory, and nonrespiratory health including asthma	Age and sex standardised rates and ratios, questionnaires (6399 adults, 1888 children) time series	Perceived exposure areas (criteria not specified), modeled exposure (model not specified) 24-hour mean daily measures of SO_2_ and smoke over 56 months (1987–91)
Aylin et al., 2001 [37]	United Kingdom	Coke works	Hospital admissions for respiratory and cardiovascular diseases	Standardised admissions rates	Distance (7.5 km)

Patel et al., 2008 [38]	India	Vapi industrial area, dyes, chemical plants	Respiratory health, lung function	Questionnaires (2, 573 women)	Distance (<2 km, 2-3 km, 3-4 km, and farther)
De Marco et al., 2010 [39]	Italy	Largest chipboard industrial park	Respiratory and skin diseases	Questionnaires (ISAAC (1998), ECRHS (2002), SIDRIA, MM040NA and MM080 standardized questionnaires, 3854 children)	Distance (no wood factories <2 km from home and school (“unexposed” group) at least 1 low emission factory (but no chipboard industries) <2 km from home or school (group “at low exposure”), at least 1 chipboard industry <2 km from home or school (group “at high exposure”)
Dubnov et al., 2007 [40]	Israel	Major coal-fired power station	Health status, pulmonary function tests (PFT), forced vital capacity (FVC) and forced expiratory volume during the first second (FEV1)	Questionnaires (ATS and National Heart and Lung Institute) (1492 children)	NO_*x*_ ∗ SO_2_ during acute episodes (NO_*x*_ and SO_2_ measurements above 0.125 and 0.070 ppm, respectively, during 30 mn), based on a map interpolated from 12 monitoring stations
Ginns and Gatrell, 1996 [41]	United Kingdom	Cement works	Respiratory health	Questionnaire (362 children)	Distance (near the industry versus area 9 to 19 km away)
Halliday et al., 1993 [42]	Australia	Power stations	Asthma, general symptoms, measurement of lung function, bronchial reactivity, and skin test atopy was	Questionnaire (851 children)	Distance (near the industry versus area 40 km away)
Peled et al., 2005 [43]	Israel	2 power plants	Health status, lung function (peak expiratory flow)	Nested cohort study (285 children), questionnaire based on the American Thoracic Society's (ATS) ATS-DLD-78	PM10 and PM2.5 daily measurements at 6 stations
Pignato et al., 2004 [44]	Italy	Petrochemical industries and oil refineries	Self-reported asthma, asthma-like symptoms, and allergic rhinitis	Questionnaires (1180 children)	Annual mean NO_2_ measurements
Rusconi et al., 2011 [45]	Italy	Biggest high complexity refinery in the Mediterranean Sea and largest European liquid fuel gasification plant	Asthma, respiratory symptoms in children, FENO, and lung function measurements	Questionnaires (ISAAC)	Measurement of weekly concentrations of SO_2_, benzene, NO_2_, O_3_
Stenlund et al., 2009 [46]	Sweden	Steel industry	Self-reported health symptoms bronchitis- and asthma-like, and neurasthenic	Interventional, population-based questionnaire study (684 adults)	distance (two areas relatively close and relatively distant)
De Moraes et al., 2010 [47]	Brazil	Petrochemical complex	Wheezing	Questionnaires (ISAAC) (209 children)	Cities in a 5-kilometer radius, communities established downwind of the petrochemical complex and thus, under greater influence of its dispersion plume (A, B, C), were classified as “exposed communities” (ECs) Those upwind of the plant and thus less exposed to its dispersion plume (D, E) were used as reference communities (RCs)
Jadsri et al., 2006 [48]	Thailand	50 chemical industries	Respiratory diseases	Spatial regression analysis	Dispersion of SO_2_, NO_*x*_, and TSP
Câra et al., 2007 [49]	Romania	Iron, steel, and coke factory	Wheezing	Comparison of two periods before and after the closure of the factory (GPs information for 874 children)	Distance (near the industry and 10 km away)
Pless-Mulloli et al., 2000 [50] Pless-Mulloli et al., 2001 [51]	United Kingdom	Opencast coal mining sites	Respiratory illnesses	Questionnaires (3216 children) and GPs records (2442 records)	Distance (5 cities near industries and 5 referent cities further away)
Smargiassi et al., 2009 [52]	Canada	Refinery	Emergency visits and hospital admissions for asthma in children	time stratified case-crossover	Distance (0.5–7.5 km) and daily SO_2_ measurements, at-home estimates of daily exposure based on a dispersion model (AERMOD)
Howel et al., 2001 [53]	United Kingdom	Opencast coal mines	Respiratory health	GP data, respiratory events (2442)	Distance, PM10 measurements
White et al., 2009 [54]	South Africa	Petrochemical refinery	Respiratory health	Questionnaire (ISAAC) (2361 children)	Distance, wind direction, and speed
Wichmann et al., 2009 [55]	Argentina	Petrochemical industries	Respiratory health, lung function (standard spirometry)	Questionnaires (1191 children)	Distance, near petrochemical industries, near heavy roads, and 2 relatively nonpolluted areas, PM and VOCs measurements
Yogev-Baggio et al., 2010[56]	Israel	Coal-fired power plant	Respiratory health, lung function (forced expiratory volume)	Questionnaires (1181 children)	NO_*x*_ ∗ SO_2_ during acute episodes (NO_*x*_ and SO_2_ measurements above 0.125 and 0.070 ppm, respectively, during 30 mn), based on a map interpolated from 12 monitoring stations
Aungudornpukdee et al., 2010 [57]	Thailand	15 chemical industries	short-term memory dysfunction	Weschsler intelligence scale for children, questionnaires (2955 children)	Distance to major air pollution sources (industries, roads, etc.)
Atari et al., 2009 [58]	Canada	Sarnia “Chemical Valley”	General health status, odour annoyance	Telephone interviews (804)	Land use regression (LUR) modeling based on SO_2_ and NO_2_ measurements

**Table 4:** Studies investigating mortality.

Reference	Country	Industrial background	Health outcome	Epidemiological design	Exposure assessment
Hodgson et al., 2007 [60]	United Kingdom	Runcorn: chlor alkali plant, power stations	Mortality from renal diseases	Standardised mortality ratio	Dispersion of mercury (ADMS)
Hodgson et al., 2004 [63]	United Kingdom	Runcorn: chlor alkali plant, power stations	Mortality, hospital admissions for kidney diseases	Standardised mortality ratio, standardized admissions rate	Distance
Dolk et al., 1999 [62]	United Kingdom	Coke work	Mortality for cardiovascular and respiratory causes	Standardised mortality ratio	Distance (2 km, 7.5 km, bands of 0.5, 1, 2, 3, 4.6, 5.7, 6.7, and 7.5 km).
Triolo et al., 2008 [64]	Italy	Industrial settlement	Mortality (all causes, cancers, cardiovascular, respiratory, diabetes, injuries, etc.)	Standardised mortality ratio	Distance: 3 concentric zones of 5 km around the industries, dispersion model (CMPM98) for SO_2_, O_3_, and SO_2_ measurements
Cambra et al., 2011 [24]	Spain	284 industries declaring the EPER emissions of pollutants	Mortality all causes, ischaemic heart disease, cerebrovascular diseases, chronic lower respiratory tract diseases	Standardised mortality ratio	Distance (<2 km, >2 km).
Sarov et al., 2008 [61]	Israel	17 plants: chemical, pharmacochemical, and heavy industry	Perinatal mortality	Standardised mortality ratio	Distance up to 20 km based on odors complaints
Bhopal et al., 1994 [12] Bhopal et al., 1998 [13]	United Kingdom	Coke ovens (66 from 1980)	Mortality	Age and sex standardised rates and ratios, Questionnaires (6399 adults, 1888 children) Time series	Perceived exposure areas (criteria not specified), modeled exposure (model not specified) 24 hour mean daily measures of SO_2_ and smoke over 56 months (1987–91)

**Table 5:** Studies investigating birth outcome.

Reference	Country	Industries	Health outcome	Method	Exposure assessment
Bhopal et al., 1994 [12]	United Kingdom	Teeside	Sex ratio, birthweights, and stillbirths	Sex ratio	Perceived exposure areas (criteria not specified), modeled exposure (model not specified)
Bentov et al., 2006 [67]	Israel	17 chemical facilities	Major congenital malformations of the central nervous system	Standardized incidence ratio	Distance (exposed < 20 km), wind direction
Brender et al., 2006 [68]	United States	113 industries in the Texas National Priority Listing (NPL) sites	Oral clefts	Logistic regression	Distances (proximity ≤ 1 mile)
Brender et al., 2008 [70]	United States	113 industries in the Texas National Priority Listing (NPL) sites	Chromosomal anomalies	Case control (2099 cases, 4368 controls)	Distances (proximity ≤ 1 mile)
Yang et al., 2000 [28]	Taiwan	Kaohsiung oil refineries	Sex ratios	Standardized sex ratio	Distance (all municipalities in the area)
Yang et al., 2002 [71]	Taiwan	Kaohsiung oil refineries	Preterm delivery	Logistic regression model	Distance (at least 50% population or 50% area falling within a distance of 3 km from any one of the three complexes)
Yang et al., 2004 [65]	Taiwan	Kaohsiung oil refineries	Preterm delivery	Logistic regression model	Distance (at least 50% population or 50% area falling within a distance of 3 km from any one of the three complexes)

**Table 6:** Biomonitoring studies.

Reference	Country	Industry	Biomarkers	*N* cases
Barregard et al., 2006 [75]	Italy and Sweden	Chlor alkali plants	Urinary mercury	193
Rusconi et al., 2011 [45]	Italy	Biggest high complexity refinery in the Mediterranean Sea and largest European liquid fuel gasification plant	MDA-dG adducts	54
Choi et al., 2000 [76]	Korea	Large-scale petrochemical industrial complex	Benzene in blood, metabolites of benzene in urine	115
Pless-Mulloli et al., 2005 [77]	United Kingdom	Teesside	Polychlorinated dibenzo-p-dioxins, furans, and polychlorinated biphenyls in blood	40
Thomas et al., 2009 [78]	United Kingdom	Large smelter lead/zinc smelter	Cadmium in urine	180
Sala et al., 1999 [79]	Spain	Organochlorine compound factory	Organochloring in blood	608
Stroh et al., 2009 [80]	Sweden	Lead smelters	Lead in blood	3879
Williamson et al., 2006 [81]	United States	Six superfund sites	Serum Immunoglobulins	3916
Thomas et al., 2009 [78]	United Kingdom	Large smelter lead/zinc smelter	Cadmium in urine	180

**Table 7:** Studies investigating mental health.

Reference	Country	Industries	Health Outcome	Method	Exposure assessment
Bush et al., 2001 [74]	United Kingdom	Teeside	Stigma	5000 questionnaires + 41 interviews	Distance (three areas at 1.5, 7, and 8 km)
Downey and Van Willigen, 2005 [73]	United States	Industries in the Toxic Release Inventory	Psychological distress (depression), perceived disorders	1210 questionnaires	Distance, visual exposure
Boardman et al., 2008 [72]	United States	Industries in the Toxic Release Inventory	psychological distress (K6 scale)	1139 questionnaires	Distance, visual exposure
